# Supplementary material for: HER2 mRNA Levels, Estrogen Receptor Activity and Susceptibility to Trastuzumab in Primary Breast Cancer
Source: Cancers (Basel). 2022 Nov 17;14(22):5650. doi: 10.3390/cancers14225650 (PMC9688101; doi:10.3390/cancers14225650)
Supplement: Supplementary file 1 [file cancers-14-05650-s001.zip › Table S1.pdf]

Table S1. Clinicopathological characteristics of HER2-positive breast carcinoma patients according to HER2 mRNA and E2 levels.

	**Cohort 1 n=36**		**Cohort 2 n=40**	
**Characteristic**	**HER2 low** n=18 (%)	**HER2 high** n=18 (%)	**E2 low** n=20 (%)	**E2 high** n=20 (%)
Age				
Median (interquartile range)	53 (47-58)	55 (50-59)	58 (56-64)	60 (57-64)
Size				
≤ 2 cm	10 (56)	9 (50)	12 (60)	15 (75)
> 2 cm	8 (44)	9 (50)	8 (40)	5 (25)
Grade				
II	4 (22)	4 (22)	6 (30)	2 (10)
III	14 (78)	14 (78)	14 (70)	18 (90)
Node[b]				
N0	3 (17)	2 (11)	9 (45)	6 (30)
N+	15 (83)	16 (89)	11 (55)	14 (70)
ER[a]				
neg	9 (50)	9 (50)	7 (35)	10 (50)
pos	9 (50)	9 (50)	13 (65)	10 (50)
PgR[a]				
neg	10 (50)	8 (60)	13 (65)	11 (55)
pos	8 (50)	10 (40)	7 (35)	9 (45)
BMI				
<20	0	0	1 (1)	0
≥20<25	3 (17)	5 (28)	15 (75)	12 (70)
≥25<30	1 (5)	0	4 (20)	4 (20)
≥30	0	0	0	2 (10)
na	14 (78)	13 (72)	0	2 (10)

[a] ER- and PgR-positive, > 10% cell positivity by immunohistochemistry

[b] Node status positive, at least one positive lymph node by histological examination
