# Supplementary figures and images for: HER2 mRNA Levels, Estrogen Receptor Activity and Susceptibility to Trastuzumab in Primary Breast Cancer
Source: Cancers (Basel). 2022 Nov 17;14(22):5650. doi: 10.3390/cancers14225650 (PMC9688101; doi:10.3390/cancers14225650)

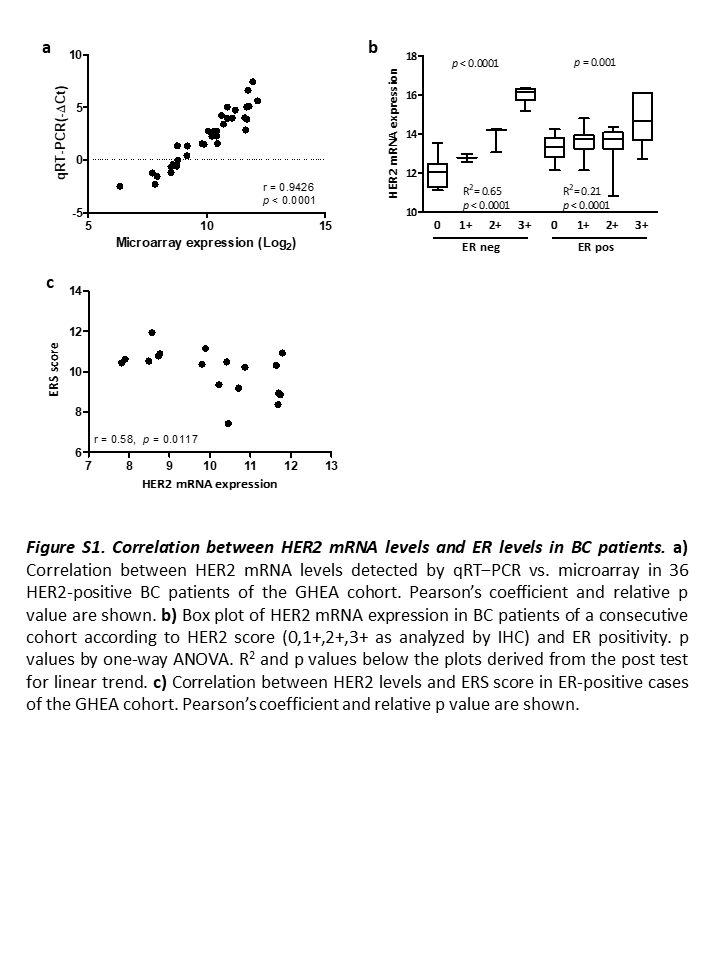

Supplement: Supplementary file 1 [file cancers-14-05650-s001.zip › Figure S1.tif]

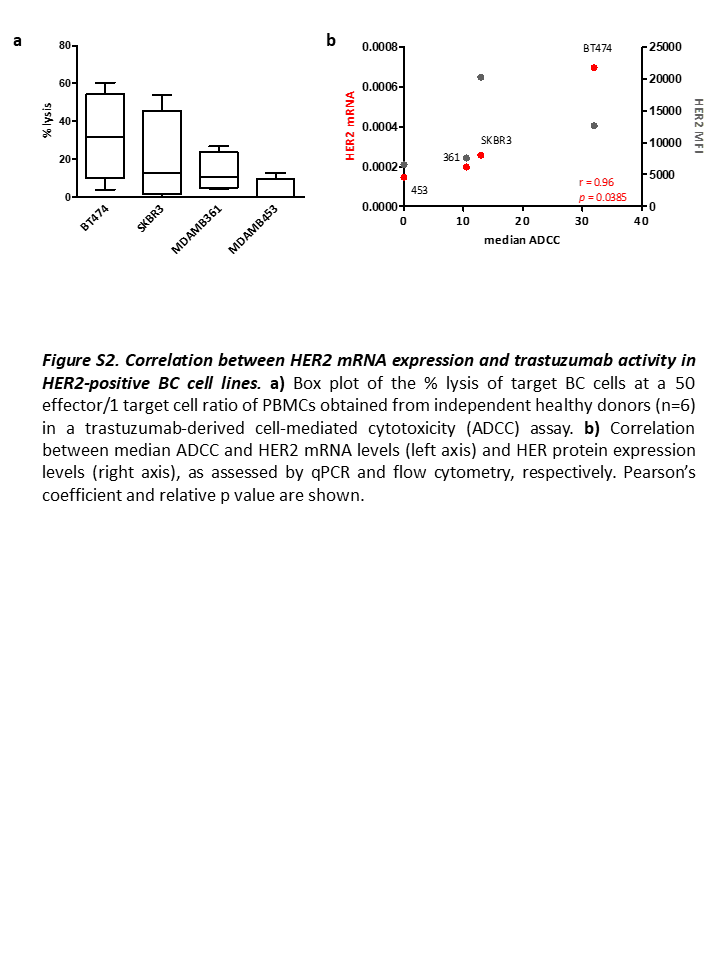

Supplement: Supplementary file 1 [file cancers-14-05650-s001.zip › Figure S2.tif]
